# Wound-Healing and Skin-Moisturizing Effects of *Sasa veitchii* Extract

**DOI:** 10.3390/healthcare9060761

**Published:** 2021-06-19

**Authors:** Nobutomo Ikarashi, Miho Kaneko, Izumi Fujisawa, Natsuko Fukuda, Ryotaro Yoshida, Risako Kon, Hiroyasu Sakai, Kiyoshi Sugiyama, Junzo Kamei

**Affiliations:** 1 Department of Biomolecular Pharmacology, Hoshi University, 2-4-41 Ebara, Shinagawa-ku, Tokyo 142-8501, Japan; miho.k-611@ezweb.ne.jp (M.K.); aqua07wing_sky48@i.softbank.jp (I.F.); s151206@hoshi.ac.jp (N.F.); s172530@hoshi.ac.jp (R.Y.); r-kon@hoshi.ac.jp (R.K.); sakai@hoshi.ac.jp (H.S.); kamei@hoshi.ac.jp (J.K.); 2 Department of Functional Molecular Kinetics, Hoshi University, 2-4-41 Ebara, Shinagawa-ku, Tokyo 142-8501, Japan; sugiyama@hoshi.ac.jp

**Keywords:** aquaporin, skin, wound healing, *Sasa veitchii*, kumazasa, mitogen-activated protein kinase, cosmetics

## Abstract

*Sasa veitchii* (*S. veitchii*) is a traditional herb derived from the bamboo genus, which is collectively called Kumazasa. Although Kumazasa extract is believed to have various effects on the skin, there is little scientific evidence for these effects. In this study, we aimed to obtain scientific evidence regarding the wound-healing and skin-moisturizing effects of Kumazasa extract. Kumazasa extract was applied to the skin of a mouse wound model for 14 days, and the wound area and dermal water content were measured. Mice treated with Kumazasa extract had smaller wound areas than control mice. The dermal water content in the Kumazasa extract-treated group was significantly higher than that in the control group. The mRNA and protein expression levels of cutaneous aquaporin-3 (AQP3), which is involved in wound healing and increases in dermal water content, were significantly increased by treatment with Kumazasa extract. Kumazasa extract-treated HaCaT cells exhibited significantly higher AQP3 expression and p38 mitogen-activated protein kinase (MAPK) phosphorylation than control cells. With continuous application, Kumazasa extract increases AQP3 expression and exerts wound-healing and moisturizing effects. The increase in AQP3 expression elicited by Kumazasa extract may be due to enhancement of transcription via activation of p38 MAPK signaling.

## 1. Introduction

The skin is an organ that separates the internal environment of an organism from the external environment and has important functions in regulating water and body temperature and protecting the body from physicochemical stimuli [1]. The outermost layer of the skin is the epidermal layer, approximately 90% of which is formed by epidermal keratinocytes [2]. When various stresses are applied to the epidermal layer, wounds form. After skin damage occurs, keratinocytes begin to proliferate actively, resulting in the formation of a hyperproliferative epithelium on the wound surface [3,4]. These events are thought to be regulated predominantly by growth factor receptors, integrins, extracellular matrix components, and matrix metalloproteinases; thus, the skin wound-healing process is a complex interplay of multiple factors [3,4].

It has recently been reported that aquaporins (AQPs) are important in skin wound healing. AQPs are channels present in the plasma membrane that transport water and glycerol [5]. In humans, 13 types (AQP0 to AQP12) have been identified and are distributed in various tissues. There are two categories of AQPs: AQPs that selectively transport only water, such as AQP0, AQP2, AQP4, and AQP5, and AQPs that transport both water and glycerol, such as AQP3, AQP7, and AQP9 [5]. Of these, AQP3 is expressed predominantly in skin keratinocytes [2,6]. It has been reported that AQP3-deficient mice have drier skin and lower skin elasticity than wild-type mice [7,8]. In addition, it has been confirmed that AQP3 in the skin is involved in cell proliferation and cell migration [9,10] and that wound healing is delayed in AQP3-deficient mice [11]. Thus, AQP3 is believed to promote wound healing by enhancing the migration and proliferation of keratinocytes.

*Sasa veitchii* (*S. veitchii*) is a traditional herb derived from the bamboo genus, which is collectively called Kumazasa. The leaves contain lignin, polysaccharides, and chlorophyll, among other components [12,13], and are used as a folk medicine for stomachaches. Recently, Kumazasa extract has been observed to have various pharmacological effects, such as antioxidant [14], anti-inflammatory [15,16], antibacterial [17,18], and anticancer effects [19], and to be useful as a health food material. In addition, Kumazasa extract has been reported to have a wound healing effect [20], and its effect on the skin is also attracting attention. However, the mechanism of wound healing improvement by Kumazasa extract is completely unknown. Moreover, other action on the skin, such as the effect of Kumazasa extract on the moisturizing action, are not known. We considered that obtaining scientific evidence is important for determining the usefulness of Kumazasa extract.

In this study, we aimed to obtain scientific evidence for the wound-healing and skin-moisturizing effects of Kumazasa extract. Specifically, a mouse wound model was created using punch biopsy, and the wound-healing and skin-moisturizing effects of topically applied Kumazasa extract were observed. In addition, we analyzed the mechanism by focusing on AQP3, which is involved in both wound-healing and skin-moisturizing functions [2,6].

## 2. Materials and Methods

### 2.1. Kumazasa Extract

Kumazasa hot water extract concentrate (Kumazasa extract) was provided by Hoshi Pharmaceutical Co., Ltd. (Tokyo, Japan). In the experiment, a mixture of equal amounts of three different lots of Kumazasa extract (H30YH1, A20YK1, and H15KG3) was used.

### 2.2. Animals

Seven-week-old HR-1 hairless mice (male) were obtained from Hoshino Laboratory Animals, Corp. (Ibaraki, Japan). The mice were used in the experiment after prebreeding for 1 week. The animals were bred in a facility with a temperature of 24 ± 1 °C, a humidity of 55 ± 5%, and lights on from 08:00 to 20:00. We carefully considered the ethical aspects of the animal experiments in our study and obtained approval (approval number: 30-125) from the animal experiment committee of Hoshi University. The protocols were conducted in accordance with the animal experiment guidelines of Hoshi University.

### 2.3. Treatments

Under isoflurane anesthesia, the skin on the back of each mouse was injured using a punch biopsy to establish a wound model [21,22]. Beginning on the day after wounding, a 50% aqueous ethanol solution or 1% Kumazasa extract solution was applied (100 μL/mouse) to the skin for 14 days, and the wound area and dermal water content were measured on the 15th day. The dermal water content was measured using a Corneometer (CM825, Courage & Khazaka, Cologne, Germany) at a temperature of 23 ± 1 °C and a humidity of 60 ± 10%. The skin was removed under isoflurane anesthesia, instantly frozen in liquid nitrogen, and then stored at −80 °C.

### 2.4. Cell Culture

The human keratinocyte cell line HaCaT (Cell Line Service, Eppelheim, Germany) was maintained in Dulbecco’s modified Eagle’s medium (DMEM) containing streptomycin, penicillin G potassium, and 10% fetal bovine serum. The HaCaT cells were seeded on plates and incubated in a CO_2_ incubator. DMEM containing Kumazasa extract (200–1000 μg/mL) was prepared and replaced with the medium of the subcultured cells. The cells were cultured for 1, 6, 24, or 72 h.

### 2.5. Real-Time RT-PCR

Total RNA was extracted from mouse skin or HaCaT cells using TRI reagent (Sigma-Aldrich Co. LLC, St. Louis, MO, USA), and cDNA was synthesized from the RNA using a High Capacity cDNA Reverse Transcription Kit (Applied Biosystems, Foster City, CA, USA). In each well of the PCR plate, SsoAdvanced Universal SYBR Green Supermix (Bio-Rad Laboratories, Hercules, CA, USA), cDNA solution, and primer solution for the target gene (Table 1) were added. The fluorescence intensity of the amplification process was monitored with a CFX Connect Real-Time System (Bio-Rad Laboratories). The mRNA expression levels of the target genes were normalized using m18s rRNA (in vivo study) or hRPL30 (in vitro study).

### 2.6. Electrophoresis and Immunoblotting

Samples were prepared from mouse skin and HaCaT cells for western blotting in the same manner as previously reported [23,24]. The protein concentration was measured by the bicinchoninic acid method using bovine serum albumin as a standard. After adding an equal amount of loading buffer (20% glycerol, 100 mM Tris, 4% sodium dodecyl sulfate, 0.004% bromophenol blue, and 10% 2-mercaptoethanol) to the sample solution, electrophoresis was performed using a polyacrylamide gel. The protein was transferred to a polyvinylidene difluoride membrane, and the membrane was blocked with skim milk solution. The membrane was then reacted with the following primary antibodies: rabbit anti-rat AQP3 (Alomone Labs, Jerusalem, Israel), mouse anti-rabbit Na/K-ATPase (Merck Millipore, Darmstadt, Germany), rabbit anti-human phospho-p38 mitogen-activated protein kinase (MAPK) (Thr180/Tyr182) (Cell Signaling Technology, Beverly, MA, USA), and rabbit anti-human p38 MAPK (Cell Signaling Technology) antibodies. After washing, the membrane was reacted with the secondary antibodies donkey anti-rabbit IgG-HRP (Santa Cruz Biotechnology Inc., Santa Cruz, CA, USA) and goat anti-mouse IgG-HRP antibody (Merck Millipore). The membrane was then reacted with ECL Prime Western Blotting Detection Reagents (GE Healthcare, Fairfield, CT, USA). The membrane was photographed with a cooled CCD camera (ImageQuant LAS500, GE Healthcare), and the band density was analyzed. The AQP3 expression levels were normalized using Na/K-ATPase expression.

### 2.7. Wound Healing Scratch Assay

The HaCaT cells were seeded on plates and incubated in a CO_2_ incubator. The confluent cells were mechanically scratched with a yellow chip. The cell debris was removed by exchanging medium. DMEM containing Kumazasa extract (1000 μg/mL) was prepared and replaced the medium of the subcultured cells. The cells were cultured for 24 or 72 h.

### 2.8. Statistical Analysis

The experimental values are shown as the mean ± standard deviation (SD). Statistics were analyzed by Student’s *t*-test or one-way analysis of variance (ANOVA) followed by Dunnett’s multiple comparison tests using using the BellCurve for Excel (Social Survey Research Information Co., Ltd., Tokyo, Japan).

## 3. Results

### 3.1. Skin Wound-Healing Effect of Kumazasa Extract

**Figure 1:**
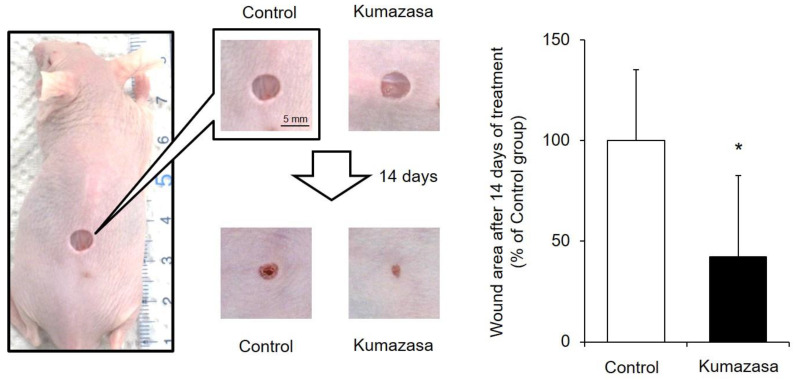
Skin wound-healing effect of Kumazasa extract. A mouse wound model was prepared, and Kumazasa extract solution was applied to the wound surface for 14 days. The wound area was measured on day 15, and the mean value of the control group is expressed as 100% (mean ± SD, *n* = 5, *: *p* < 0.05 vs. control group).

After Kumazasa extract was applied to a murine cutaneous wound model for 14 days, the wound area was analyzed (Figure 1). The skin wound area on day 15 in the Kumazasa extract-treated group was significantly smaller than that in the control group.

### 3.2. Skin-Moisturizing Effect of Kumazasa Extract

**Figure 2:**
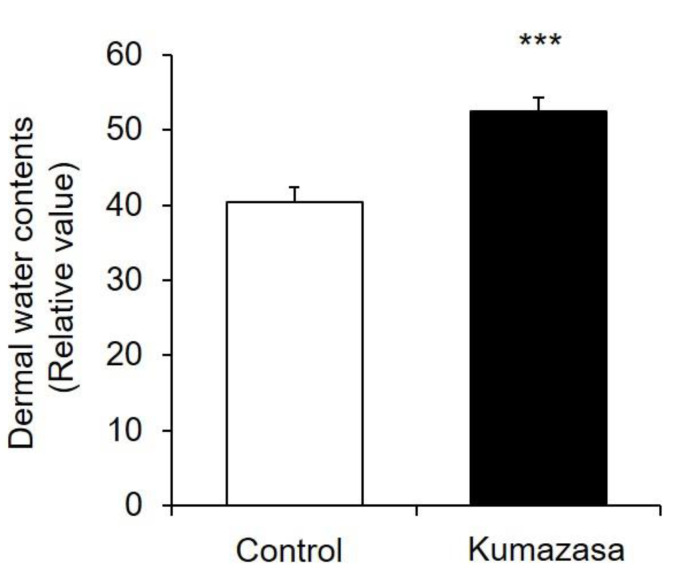
Skin-moisturizing effect of Kumazasa extract. Kumazasa extract solution was applied to the skin of mice for 14 days, and the dermal water content was measured on the 15th day (mean ± SD, *n* = 5, ***: *p* < 0.001 vs. control group).

Kumazasa extract was applied to the skin of mice for 14 days, and the dermal water content was measured (Figure 2). The dermal water content on the 15th day in the Kumazasa extract-treated group was significantly (approximately 20%) higher than that in the control group.

### 3.3. AQP3 Expression Level in Mouse Skin

**Figure 3:**
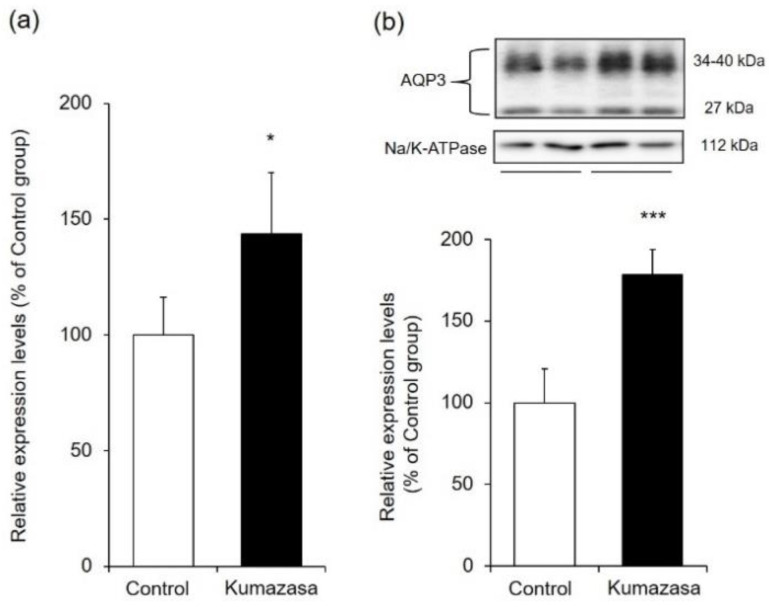
AQP3 expression level in mouse skin. Kumazasa extract solution was applied to the skin of mice for 14 days, and the skin was removed on the 15th day. The mRNA (**a**) and protein (**b**) expression levels of AQP3 were analyzed by real-time RT-PCR and western blotting, respectively (mean ± SD, *n* = 5, *: *p* < 0.05, ***: *p* < 0.001 vs control group).

AQP3 is highly expressed in the skin and is involved in cell migration and intercellular water transport. Therefore, we investigated whether changes in the expression of cutaneous AQP3 are involved in the wound-healing and skin-moisturizing effects of Kumazasa extract (Figure 3).

The mRNA expression level of AQP3 was analyzed by real-time RT-PCR and was found to be approximately 1.4 times higher in the Kumazasa extract-treated group than in the control group.

When the protein expression level of AQP3 was analyzed by western blotting, bands of AQP3 were detected at approximately 27 kDa and approximately 30–40 kDa. It has been reported that these bands are nonglycosylated AQP3 (27 kDa) and glycosylated AQP3 (30–40 kDa), respectively [25,26]. Although these two forms exhibit differences in AQP stability and the amount of transfer to the cell membrane, their water permeability does not differ [27,28,29]. Therefore, in this study, the levels represented by these bands were added to determine the total AQP3 expression level. The protein expression level of cutaneous AQP3 in the Kumazasa extract-treated group was significantly (approximately 1.6 times) higher than that in the control group.

### 3.4. Expression Levels of Function-Regulating Genes in Mouse Skin

**Figure 4:**
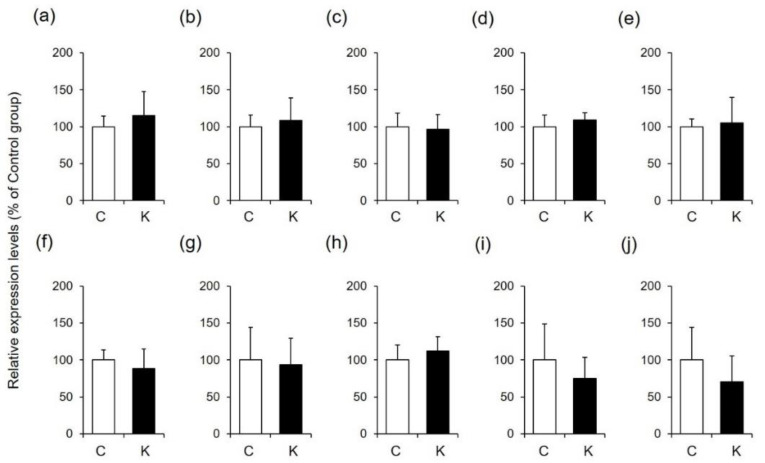
Expression levels of function-regulating genes in mouse skin. A 50% ethanol solution (C) or Kumazasa extract solution (K) was applied to the skin of mice for 14 days, and the skin was removed on the 15th day. The mRNA expression levels of filaggrin (**a**), loricrin (**b**), Has2 (**c**), Hyal1 (**d**), Sptlc1 (**e**), Sptlc2 (**f**), Acer1 (**g**), Asah1 (**h**), Col1a1 (**i**), and Col1a2 (**j**) were analyzed by real-time RT-PCR (mean ± SD, *n* = 5).

Skin function is controlled by various factors, such as filaggrin, loricrin, hyaluronic acid, ceramide, and collagen [30,31,32]. Since changes in the amounts of these factors are involved in wound healing and skin moisturization, we hypothesized that Kumazasa extract may affect factors other than AQP3. Therefore, we analyzed the expression levels of genes associated with these skin function regulators (Figure 4).

The mRNA expression levels of filaggrin and loricrin in the skin were not changed by application of Kumazasa extract. In addition, the expression levels of enzymes involved in the degradation and synthesis of hyaluronic acid and ceramide were not affected by treatment with Kumazasa extract. Furthermore, there was no difference in the amount of collagen between the control group and the Kumazasa extract-treated group.

### 3.5. AQP3 Expression Level in HaCaT Cells

**Figure 5:**
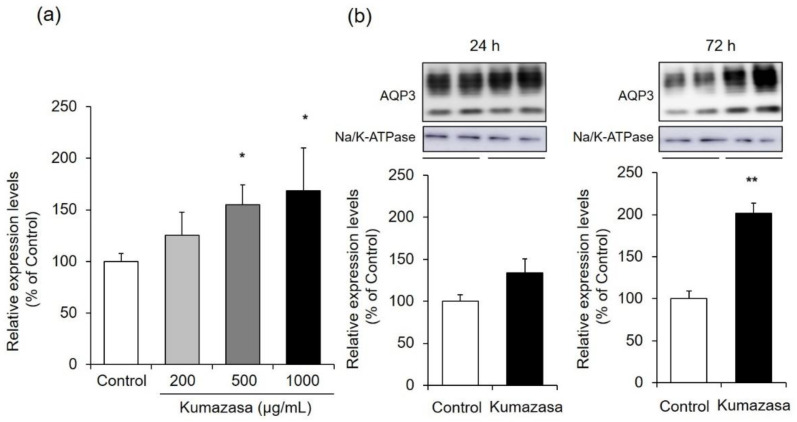
AQP3 expression level in HaCaT cells. (**a**) HaCaT cells were treated with Kumazasa extract (200–1000 µg/mL) for 6 h, and the mRNA expression level of AQP3 was analyzed by real-time RT-PCR. (**b**) HaCaT cells were treated with Kumazasa extract (1000 µg/mL) for 24 or 72 h, and the protein expression level of AQP3 was analyzed by western blotting (mean ± SD, *n* = 4, *: *p* < 0.05, **: *p* < 0.01 vs. control cells).

We also investigated whether the Kumazasa extract-induced increase in AQP3 expression observed in mouse skin also occurred in HaCaT cells, which are human epidermal keratinocytes (Figure 5).

Six hours of treatment with Kumazasa extract increased the mRNA expression level of AQP3 in treated HaCaT cells compared with control cells in a concentration-dependent manner. In particular, significant differences were observed at Kumazasa extract concentrations of 500 μg/mL and higher.

The protein expression level of AQP3 showed an increasing tendency 24 h after the addition of Kumazasa extract, although there was not a significant difference between the treated cells and the control cells. In contrast, 72 h after the addition of Kumazasa extract, the AQP3 protein level was significantly (approximately two times) higher in treated cells than in control cells.

### 3.6. Wound-Healing Effect of Kumazasa Extract in HaCaT Cells

**Figure 6:**
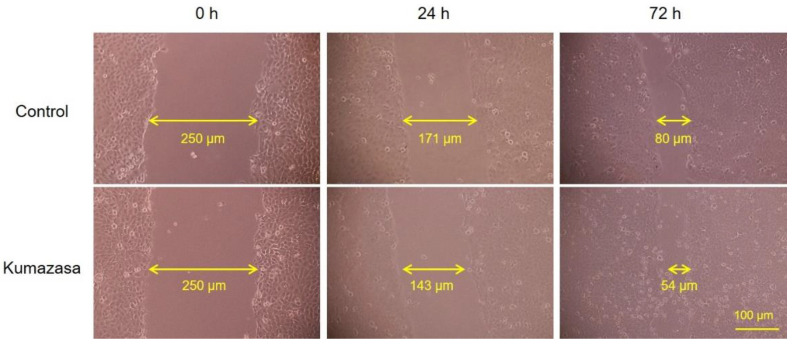
Wound-healing effect of Kumazasa extract in HaCaT cells. Kumazasa extract (1000 µg/mL) was added to the mechanically scratched HaCaT cells, and the cells was observed under a microscope.

A scratch assay was used to observe whether wound healing could be confirmed at the concentration of Kumazasa extract, which showed increased expression of AQP3 in HaCaT cells. As a result, it was confirmed that the addition of Kumazasa extract enhances wound healing in HaCaT cells as compared with the control (Figure 6).

### 3.7. Expression Level of Phospho-p38 MAPK

**Figure 7:**
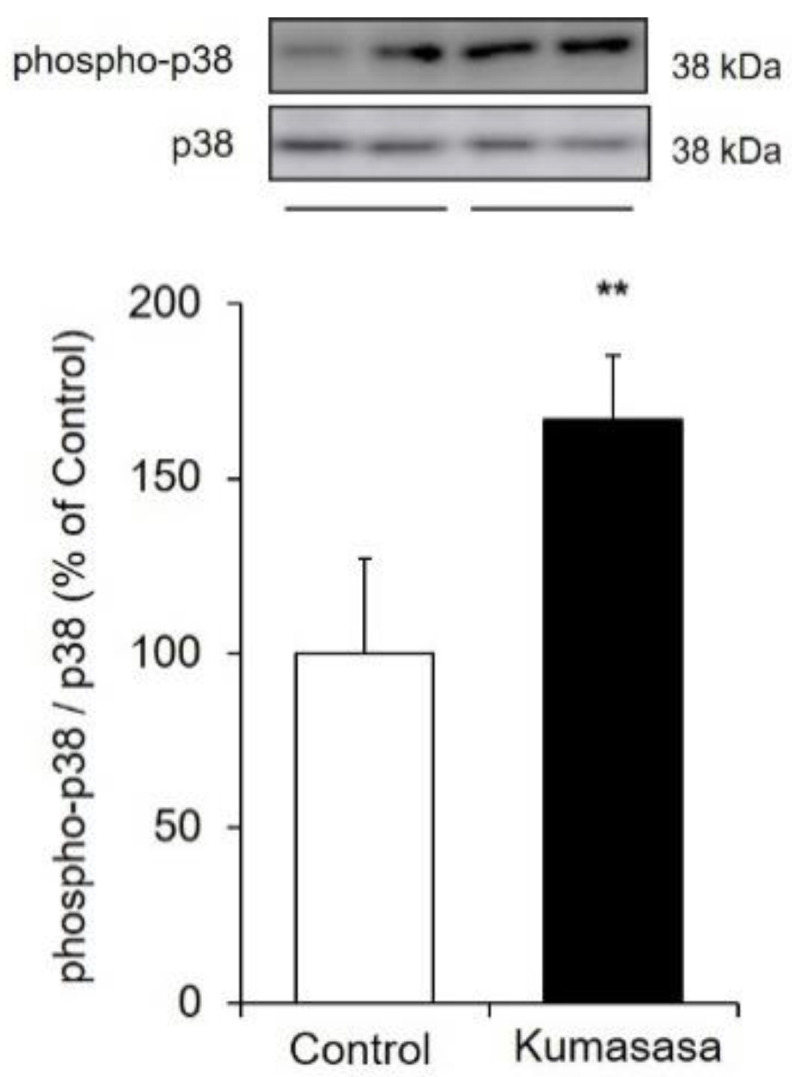
Expression level of phospho-p38 MAPK. HaCaT cells were cultured with Kumazasa extract (1000 µg/mL) for 1 h, and the protein expression levels of p38 MAPK and phospho-p38 MAPK were analyzed by western blotting (mean ± SD, *n* = 4, **: *p* < 0.01 vs. control cells).

MAPK signaling pathways consisting of p38 MAPK, extracellular signal-regulated kinase 1/2 (ERK1/2) and c-Jun NH_2_-terminal kinase 1/2 (JNK1/2) regulate the expression of various genes. Among these components, p38 MAPK has been shown by various studies to be involved in wound healing [33,34,35] and to be part of an important regulatory mechanism for AQP3 expression [11,36]. Therefore, we investigated the mechanism by which Kumazasa extract increased AQP3 expression, focusing on the activation of p38 MAPK signaling (Figure 7).

Kumazasa extract was added to HaCaT cells, and the expression levels of total p38 MAPK and phospho-p38 MAPK were analyzed to evaluate the degree of phosphorylation. The expression level of phospho-p38 MAPK was significantly increased (by approximately 1.7 times) by treatment with Kumazasa extract.

## 4. Discussion

A mouse wound model was prepared with previously described methods [21,22], and the wound-healing effect of Kumazasa extract was investigated. We found that the wound area was significantly smaller in the group treated with Kumazasa extract for 14 days than in the control group (Figure 1). In addition, treatment with Kumazasa extract increased the dermal water content (Figure 2). The above findings suggest that Kumazasa extract has a wound-healing effect and a skin-moisturizing effect.

AQP3 is expressed in the basal layer of the skin and is involved in the transport of water and glycerol. It has been reported that wound healing is delayed in AQP3-deficient mice due to decreased proliferation of keratinocytes and that this decreased proliferation is reversed by supplementation with glycerol [11]. It has also been suggested that AQP3 expression is decreased in the skin of diabetic rats, which is associated with impaired wound healing during diabetes [37]. On the other hand, the dermal water content and skin elasticity are significantly reduced in AQP3-deficient mice [7,8]. In addition, lower skin AQP3 levels are observed in old mice than in young mice, suggesting that AQP3 loss is involved in skin dryness in old age [38]. Cutaneous AQP3 levels are also decreased at the onset of diabetes mellitus [23,39], vitiligo [40,41], and psoriasis [42], in which dry skin is observed. Moreover, skin disorders such as dry skin caused by epidermal growth factor receptor inhibitor administration may also be associated with decreased skin expression of AQP3 [24]. Thus, AQP3 is considered to be a key molecule that plays important roles in wound healing and skin moisturization. Therefore, we focused on AQP3 to analyze the mechanisms of the wound-healing and skin-moisturizing effects of Kumazasa extract. Both the mRNA and protein expression levels of AQP3 in the skin were significantly higher in the mice to which Kumazasa extract was applied than in the control mice (Figure 3). In addition, in HaCaT cells, which are human epidermal keratinocytes commonly used for AQP3 research (including research seeking to identify substances that increase the expression of AQP3), Kumazasa extract increased the mRNA expression level of AQP3 in a concentration-dependent manner (Figure 5a). Moreover, treatment of HaCaT cells with Kumazasa extract in the culture medium for 72 h increased the protein expression level of AQP3 (Figure 5b). In addition, in the scratch assay using HaCaT cells, Kumazasa extract showed a wound healing effect (Figure 6). It was also found that the Kumazasa extract has a proliferative effect on HaCaT cells (Figure S1). These findings reveal that Kumazasa extract effectively increases AQP3 expression and that this effect may be involved in the wound-healing and skin-moisturizing effects of the extract. The degree of the increase in AQP3 protein expression in HaCaT cells was smaller after 24 h of treatment than after 72 h of treatment with Kumazasa extract (Figure 5b), suggesting that this effect of the extract requires continuous stimulation. However, after Kumazasa extract was orally administered to mice for 14 days, the AQP3 expression level did not differ between the Kumazasa extract-treated mice and the control mice (data not shown). Although direct skin application and oral administration of Kumazasa extract result in different concentrations of the extract in the skin, the greater effectiveness of topical application is consistent with the expectation that the extract affects the skin.

The expression level of AQP3 is regulated by the MAPK pathway members p38 MAPK, ERK1/2, and JNK1/2 [43]. Of these three major MAPK pathway members, p38 MAPK is significantly involved in wound healing. For example, it has been reported that (1) transforming growth factor-β (TGF-β), one of the important growth factors for wound healing, regulates p38 MAPK signaling in human peritoneal mesothelial cells to increase AQP3 expression [36]; that (2) mitogen-induced cell proliferation is disturbed in AQP3-deficient keratinocytes, which exhibit reduced p38 MAPK activity [11]; and that (3) p38 MAPK signaling is involved in the increase in AQP3 expression induced by agerarin from *Ageratum houstonianum* [44]. In the current study, the phosphorylation of p38 MAPK was confirmed to be enhanced in Kumazasa extract-treated HaCaT cells compared with control cells (Figure 7). Therefore, activation of p38 MAPK may be involved in one of the mechanisms by which Kumazasa extract increases the expression of AQP3. Since it is also known that transcription of AQP3 is enhanced by protein kinase A or protein kinase C activators [45,46] and peroxisome proliferator-activated receptor-γ agonists [47,48], further multifaceted analysis is required to discover the active component and to elucidate its expression regulation mechanism.

Various factors in the skin are involved in wound healing and skin moisturization [30,31,32]. Our analysis of the expression levels of function-regulating genes in mouse skin revealed that Kumazasa extract application did not affect filaggrin, loricrin, hyaluronic acid synthase 2 (Has2), hyaluronic acid-degrading enzyme (hyaluronidase 1; Hyal1), ceramide synthase (serine palmitoyltransferase 1; Sptlc1, serine palmitoyltransferase 2; Sptlc2), ceramide-degrading enzyme (alkaline ceramidase 1; Acer1, N-acylsphingosine amidohydrolase 1; Asah1), or type I collagen (collagen type I alpha 1 chain; Col1a1, collagen type I alpha 2 chain; Col1a2) expression levels (Figure 4). Therefore, AQP3 may be involved in the main mechanism underlying the wound-healing and skin-moisturizing effects of Kumazasa extract.

In a preliminary study, we investigated the effect of different lots of Kumazasa extract on AQP3 expression. Although there was a slight difference in the degree of activity, a concentration-dependent effect of increasing the AQP3 expression was observed in all lots. Based on these results, we decided to evaluate the average action of Kumazasa extract. Therefore, we conducted the study with a mixture of equal amounts of three different lots of Kumazasa extract, not a single lot.

From the above results, although the mechanism by which Kumazasa extract increases cutaneous AQP3 expression was presumed, detailed analysis requires studies using knock-down and overexpression systems. We are also interested in the effects of Kumazasa extract on ERK1/2 and JNK1/2, which are involved in the regulation of AQP3 expression, as well as p38 MAPK. Clarifying these points, including the search for active ingredients, may reveal new effects of Kumazasa extract.

The results of this study suggest that continuously applied Kumazasa extract increases cutaneous AQP3 expression and exerts wound-healing and moisturizing effects. In addition, the AQP3-increasing effect of Kumazasa extract may be due to enhancement of AQP3 transcription due to activation of p38 MAPK signaling. Since the AQP3 expression-increasing effect of Kumazasa extract was observed not only in mouse skin but also in human epidermal keratinocytes, this effect may occur in humans as well. Currently, active searches are underway for substances that increase the expression of AQP3, which is a functional molecule in the skin [49,50]. In the future, development of adhesive plasters and cosmetics containing Kumazasa extract that have AQP3-increasing effects can be expected to support new strategies for wound healing and alleviation of dry skin.

## 5. Conclusions

In conclusion, the results of this study suggest that with continuous application, Kumazasa extract increases AQP3 expression and exerts wound-healing and moisturizing effects. The increase in AQP3 expression elicited by Kumazasa extract may be due to enhancement of transcription via activation of p38 MAPK signaling.

## Figures and Tables

**Table 1:** Primer sequences.

Gene	Forward (5′ to 3′)	Reverse (5′ to 3′)
mAqp3	AGACAGCCCCTTCAGGATTT	TCCCTTGCCCTGAATATCTG
mFilaggrin	AAGGAAATCAGTCTTGCCGT	CTGACCTTCTGAGACACACC
mLoricrin	GCCGATGGGCTTAACTTTCT	CAGGATACACCTTGAGCGAC
mAcer1	CCGAGTTCTACAATACGTTCA	CATACGGATGCATGAGGAAC
mAsah1	CTGTCCTCAACAAGCTGACTG	TCTCAGTACGTCCTCAAGGC
mSptlc1	TCCCCTTCCAGAACTGGTTAAA	CCATAGTGCTCGGTGACT
mSptlc2	GTCAGGAAATTGGAAACCTGG	AGCTTCCACACCTAAGAACC
mHyal1	TTTCTTTGAGCCTGGAGCTA	GTAGTTTCCTTTCGTTGGCT
mHas2	CGTGGATTATGTACAGGTGTGT	CCAACACCTCCAACCATAGG
mCol1a1	CCCGAGGTATGCTTGATCTG	GGTGATACGTATTCTTCCGGG
mCol1a2	TCTCACTCCTGAAGGCTCTA	GTAGTAATCGCTGTTCCACTC
m18S rRNA	GTCTGTGATGCCCTTAGATG	AGCTTATGACCCGCACTTAC
hAQP3	AGACAGCCCCTTCAGGATTT	TCCCTTGCCCTGAATATCTG
hRPL30	GAAGACGAAAAAGTCGCTGG	GACCAATTTCGCTTTGCCTT

## Data Availability

Not applicable.

## Supplementary Materials

The following are available online at https://www.mdpi.com/article/10.3390/healthcare9060761/s1, Figure S1: Effects of Kumazasa extract on cell proliferation in HaCaT cells.
