# Supplementary material for: Wound-Healing and Skin-Moisturizing Effects of Sasa veitchii Extract
Source: Healthcare (Basel). 2021 Jun 19;9(6):761. doi: 10.3390/healthcare9060761 (PMC8235400; doi:10.3390/healthcare9060761)
Supplement: Supplementary file 1 [file healthcare-09-00761-s001.zip › healthcare-1235216-SI.pdf]

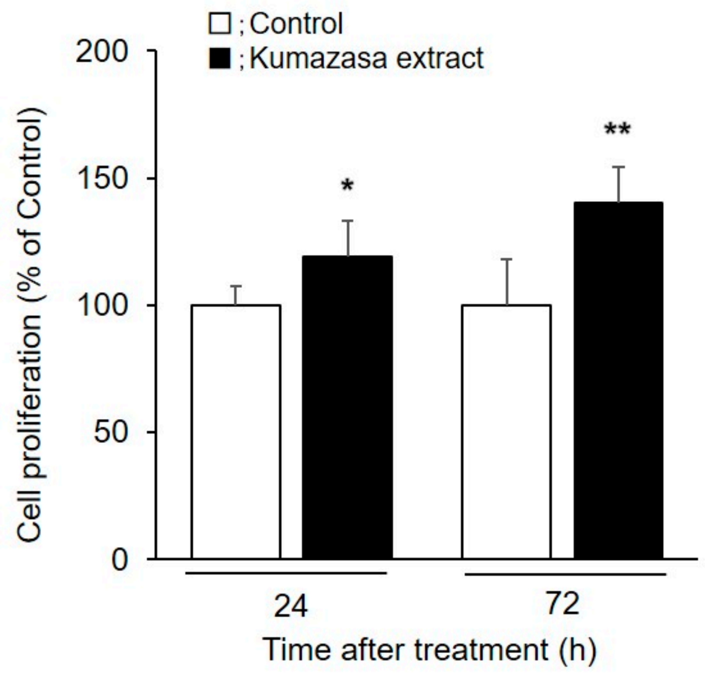

Figure S1. Effects of Kumazasa extract on cell proliferation in HaCaT cells. HaCaT cells were plated and incubated for 24 h or 72 h after the addition of Kumazasa extract (1000 μg/mL). Cell proliferation was analyzed by the WST-1 assay. The mean value of the control cells is expressed as 100% (mean ± SD, n=6, *: p <0.05, **: p <0.01 vs control cells)
